# Awake prone positioning in patients with hypoxemic respiratory failure due to COVID-19: the PROFLO multicenter randomized clinical trial

**DOI:** 10.1186/s13054-021-03602-9

**Published:** 2021-06-14

**Authors:** Jacob Rosén, Erik von Oelreich, Diddi Fors, Malin Jonsson Fagerlund, Knut Taxbro, Paul Skorup, Ludvig Eby, Francesca Campoccia Jalde, Niclas Johansson, Gustav Bergström, Peter Frykholm, the PROFLO Study Group, Anna Gradin, Mustafa Ali, Ulrica Lennborn, Darko Bogdanovic, Andreas Roos, Matilda Modie, Julia Giesecke

**Affiliations:** 1 Department of Surgical Sciences, Section of Anaesthesiology and Intensive Care Medicine, Uppsala University, Entrance 78, 1 floor, 751 85 Uppsala, Sweden; 2 Perioperative Medicine and Intensive Care, Karolinska University Hospital, Solna, Sweden; 3 Department of Physiology and Pharmacology, Section of Anesthesiology and Intensive Care Medicine, Karolinska Institutet, Solna, Sweden; 4 Department of Anaesthesiology and Intensive Care Medicine, Ryhov County Hospital, Jönköping, Sweden; 5 Department of Medical Sciences, Section of Infectious Diseases, Uppsala University, Uppsala, Sweden; 6 Acute and Reparative Medicine, Karolinska University Hospital, Solna, Sweden; 7 Department of Molecular Medicine and Surgery, Section of Thoracic Anesthesiology and Intensive Care, Karolinska Institutet, Solna, Sweden; 8 Department of Infectious Diseases, Karolinska University Hospital, Solna, Sweden; 9 Infectious Diseases Unit, Department of Medicine, Karolinska Institutet, Solna, Sweden

**Keywords:** COVID-19, Awake prone positioning, Intensive care, Critical care, Respiratory failure, High-flow nasal oxygen, Noninvasive ventilation, Intubation rates, Mechanical ventilation

## Abstract

**Background:**

The effect of awake prone positioning on intubation rates is not established. The aim of this trial was to investigate if a protocol for awake prone positioning reduces the rate of endotracheal intubation compared with standard care among patients with moderate to severe hypoxemic respiratory failure due to COVID-19.

**Methods:**

We conducted a multicenter randomized clinical trial. Adult patients with confirmed COVID-19, high-flow nasal oxygen or noninvasive ventilation for respiratory support and a PaO_2_/FiO_2_ ratio ≤ 20 kPa were randomly assigned to a protocol targeting 16 h prone positioning per day or standard care. The primary endpoint was intubation within 30 days. Secondary endpoints included duration of awake prone positioning, 30-day mortality, ventilator-free days, hospital and intensive care unit length of stay, use of noninvasive ventilation, organ support and adverse events. The trial was terminated early due to futility.

**Results:**

Of 141 patients assessed for eligibility, 75 were randomized of whom 39 were allocated to the control group and 36 to the prone group. Within 30 days after enrollment, 13 patients (33%) were intubated in the control group versus 12 patients (33%) in the prone group (HR 1.01 (95% CI 0.46–2.21), *P* = 0.99). Median prone duration was 3.4 h [IQR 1.8–8.4] in the control group compared with 9.0 h per day [IQR 4.4–10.6] in the prone group (*P* = 0.014). Nine patients (23%) in the control group had pressure sores compared with two patients (6%) in the prone group (difference − 18% (95% CI − 2 to − 33%); *P* = 0.032). There were no other differences in secondary outcomes between groups.

**Conclusions:**

The implemented protocol for awake prone positioning increased duration of prone positioning, but did not reduce the rate of intubation in patients with hypoxemic respiratory failure due to COVID-19 compared to standard care.

***Trial registration*:**

ISRCTN54917435. Registered 15 June 2020 (https://doi.org/10.1186/ISRCTN54917435).

**Supplementary Information:**

The online version contains supplementary material available at 10.1186/s13054-021-03602-9.

## Introduction

Prone positioning reduces mortality in intubated and mechanically ventilated patients with moderate to severe acute respiratory distress syndrome (ARDS) [1, 2]. Awake prone positioning (APP) in non-intubated, spontaneously breathing patients with hypoxemic respiratory failure has gained wide-spread use in health care systems overwhelmed by patients with Coronavirus disease 2019 (COVID-19) [3–5] although previously rarely reported [6–9].

Prone positioning improves respiratory mechanics and gas exchange owing to several mechanisms in non-intubated spontaneously breathing and intubated mechanically ventilated patients. It increases lung volume [10, 11], improves ventilation-perfusion ratio [12–14] and distributes pleural pressure more evenly [15]. Several studies report transient improvement in oxygenation during APP in a majority of patients with hypoxemic respiratory failure due to COVID-19 pneumonia [3, 16–23]. However, translating physiological improvement into clinically relevant outcomes has not been supported by ARDS-studies [24] and there remains a gap in the current knowledge for the use of APP [25–28]. To date, the effect of APP on intubation rates in patients with hypoxemic respiratory failure has not been studied in a randomized clinical trial.

The primary aim of this trial was to determine if a protocol for APP and standard care reduces the rate of endotracheal intubation compared to standard care alone among COVID-19 patients with hypoxemic respiratory failure supported with high-flow nasal oxygen (HFNO) or noninvasive ventilation (NIV).

## Materials and methods

### Trial design and study setting

We conducted a prospective multicenter, open-label, parallel arm, randomized clinical superiority trial in accordance with the 1964 Helsinki Declaration, Good Clinical Practice and the Consolidated Standards of Reporting Trials (CONSORT) guidelines. The trial was conducted at two tertiary teaching hospitals and one county hospital in Sweden between October 7, 2020, and February 7, 2021; 30-day follow-up was complete March 9, 2021. The trial protocol was prospectively registered at the ISRCTN registry (ISRCTN54917435) June 15, 2020 (http://isrctn.com/). Ethical approval (2020-02743) was provided by the Swedish Ethical Review Authority June 10, 2020. Written informed consent was obtained from all subjects. The trial was overseen by a trial steering committee and an independent data and safety monitoring board.

### Patients

Adults (≥ 18 years old) with COVID-19 verified by positive SARS-CoV-2 reverse transcription polymerase chain reaction tests on naso- or oropharyngeal swabs and hypoxemic respiratory failure, HFNO or NIV for respiratory support and a PaO_2_/FiO_2_-ratio ≤ 20 kPa or corresponding values of SpO_2_ and FiO_2_ (Additional file 1: eTable 1) for more than one hour, were eligible for inclusion.

Exclusion criteria were the following: oxygen supplementation with a device other than HFNO or NIV; inability to assume prone or semi-prone position; immediate need for endotracheal intubation; severe hemodynamic instability; previous intubation for COVID-19 pneumonia; pregnancy; terminal illness with less than one year life expectancy; do-not-intubate order; inability to understand oral or written study information.

#### Randomization and masking

Randomization was performed with an allocation ratio of 1:1 and a block size of eight. Randomization allocation was obtained via a centralized web-based system. Due to the nature of the intervention, the patient, the treating physician, care providers, data collectors and outcome assessors were aware of the allocation.

#### Trial protocol

After enrollment by members of the research team, patients were randomly assigned to one of two groups (Additional file 1: eFigure 1):

1. *Control group.* APP was not encouraged but could be prescribed by the attending clinician at his/her discretion.
2. *Prone group.* A protocol targeting at least 16 h APP per day was initiated. Prone and semi-prone positioning was allowed (Additional file 1: eFigure 2a-c). Flat supine positioning was discouraged and patients were instructed to place themselves in the semi-recumbent or lateral position in between proning sessions. During in-hospital transportation, oxygenation by face mask and positioning appropriate for adequate monitoring and safety was allowed.

Protocol discontinuation criteria were intubation, death or clinical improvement defined as the use of standard nasal cannula or open face mask with an oxygen flow rate of ≤ 5 L min^−1^ for 12 h. Attending clinicians could withdraw the patient from the trial at any time if they considered APP unsafe.

#### Standard care

Standard care was delivered in both groups according to clinical practice in participating hospitals. Intravenous sedation was allowed but not protocolized. Decision to intubate was made at the discretion of the attending clinician but followed local guidelines. Positioning after intubation was not protocolized, but liberal prone positioning was part of the clinical routine for mechanically ventilated patients with COVID-19 fulfilling criteria for moderate to severe ARDS [29] at all three centers.

#### Data collection

Data on age, sex, weight, height, comorbidities, location of enrollment (ward or ICU), PaO_2_, SpO_2_, FiO_2_ and respiratory rate were recorded at the time of enrollment. Positive-end expiratory pressure was recorded if the patient had NIV for respiratory support. APP duration was recorded continuously by health care providers on case report forms or in electronic data monitoring systems as available. Intubation and use of NIV, continuous renal replacement therapy (CRRT), vasopressor/inotropic support and extracorporeal membrane oxygenation (ECMO) were recorded daily. Data quality and compliance to Good Clinical Practice was verified by independent reviewers. Anonymized data were entered in a secure electronic case report form (OpenClinica®, OpenClinica LLC, Waltham, MA, USA).

#### Outcome measures

The primary endpoint was intubation within 30 days after enrollment. Secondary endpoints were duration of APP, use of NIV and time to NIV for patients included with HFNO, use of vasopressors/inotropes, CRRT, ECMO, ventilator-free days, days free of NIV/HFNO for patients not intubated, hospital and ICU length of stay, 30-day mortality, WHO-ordinal scale for clinical improvement [30] at day 7 and 30, and adverse events. Ventilator-free days were calculated for intubated patients and defined as days free from invasive mechanical ventilation from enrollment until day 30. If the patients died, zero ventilator-free days were registered.

#### Sample size calculation

Sample size calculation was based on previous studies [31, 32]. Assuming an intubation rate of 88% in the control group, we estimated a sample size of 224 patients to detect a 20% decrease of intubation in the prone group with 90% power at a type I error rate of 5%. To compensate for patients withdrawing consent, 240 patients were planned for inclusion.

#### Statistical methods

An interim analysis was planned a priori when half of the patients had been included. The decision to terminate the trial could be based on futility, safety or efficacy (Additional file 1: eTable 2). Due to rapidly declining case numbers in January 2021, the interim analysis was performed when 75 patients had been included in the study. Following the blinded analysis by the data and safety monitoring board, a re-estimation of the sample size was performed based on the observed event rate. To detect a decrease in intubation rate of 20% a sample size > 2000 patients would be required, or > 8000 patients to detect a 10% decrease in intubation rate. Therefore, early trial termination due to futility was decided.

The analyses were performed on an intention-to-treat basis. Continuous variables were reported as median (interquartile range [IQR]). Categorical variables were expressed as numbers and percentages. The primary endpoint, intubation within 30 days was analyzed using Kaplan–Meier survival analysis and compared between groups with Cox’s proportional-hazards model. Mann–Whitney *U*-test was used to compare non-normally distributed variables. Categorical variables were compared using *χ*^2^-test or Fisher’s exact test. We did not correct for multiple statistical testing in the analyses of secondary and exploratory endpoints. Two-sided *P*-values < 0.05 were considered statistically significant. Statistical analyses were performed using R Statistical Software.

## Results

### Patient characteristics

**Fig. 1:**
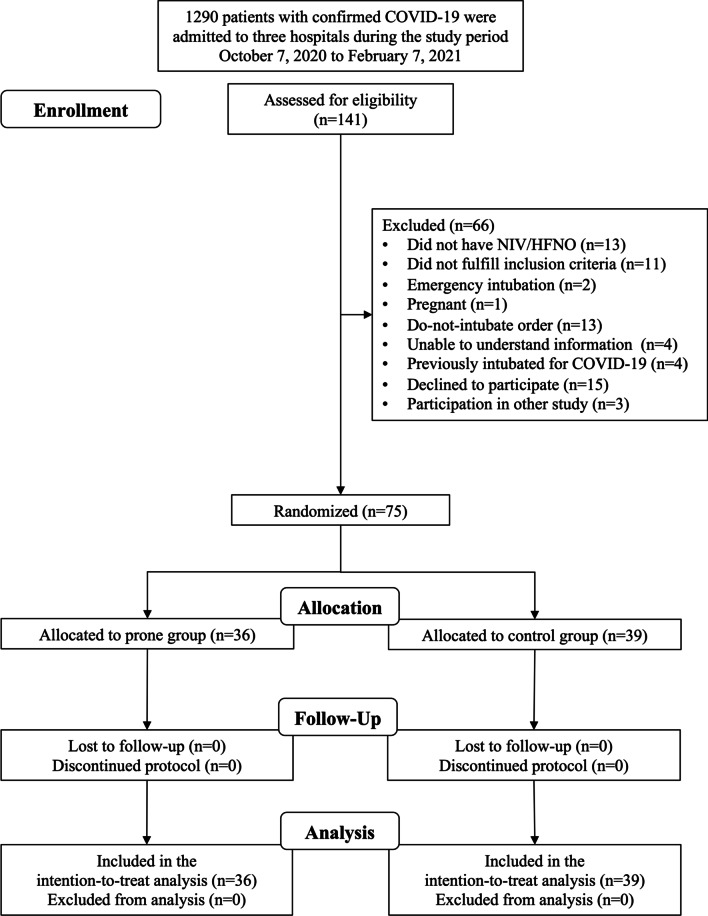
Consolidated standards of reporting trials (CONSORT) flow diagram of randomized and analyzed participants

From October 7, 2020, through February 7, 2021, 1290 patients with confirmed COVID-19 were admitted to the three participating hospitals. One hundred and forty one patients were screened, of whom 75 were randomized (Fig. 1). No patients were lost to follow-up or withdrew consent. End of follow-up was March 9, 2021. Hypertension, diabetes, obesity and lung disease were the most common comorbidities (Table 1).

**Table 1:** General characteristics of the study cohort at inclusion

Variable	Control group	Prone group
Count	39	36
Male	32 (82%)	23 (64%)
Age	65 [55–70]	66 [53–74]
BMI	29 [27–33]	28 [25–30]
Obesity (BMI ≥ 30 kg m^−2^)	12 (32%)	8 (23%)
Hypertension	21 (55%)	17 (47%)
Ischemic cardiac disease	5 (13%)	6 (17%)
Congestive heart failure	6 (15%)	2 (6%)
Lung disease	10 (26%)	4 (11%)
Asthma	5 (13%)	1 (3%)
COPD	4 (10%)	2 (6%)
Fibrosis	0 (0%)	1 (3%)
Sarcoidosis	1 (3%)	0 (0%)
Diabetes mellitus	11 (28%)	14 (39%)
Renal disease^a^	2 (5%)	3 (8%)
Active cancer	1 (3%)	4 (11%)
Liver disease	1 (3%)	0 (0%)
Enrollment outside ICU	20 (51%)	19 (53%)
HFNO	29 (74%)	31 (86%)
Flow rate (HFNO)	50 [40–50]	50 [40–50]
PEEP (NIV)	8 [6–8]	7 [6–10]
FiO_2_	0.6 [0.55–0.70]	0.6 [0.55–0.70]
SpO_2_	94 [92–95]	93 [91–94]
PaO_2_	9.2 [8.2–10]	8.8 [7.7–9.7]
RR	26 [23–32]	24 [21–29]
PaO_2_/FiO_2_ ratio	15.4 [12.5–17.3]	15.4 [11.5–17.4]
SpO_2_/FiO_2_ ratio	157 [136–175]	151 [131–174]
SBP	130 [120–140]	130 [120–140]
DBP	70 [60–80]	69 [62–75]

Categorical parameters are presented as *n* (%), continuous variables as median (interquartile range [IQR]); COPD, Chronic obstructive pulmonary disease; BMI, Body Mass Index; ICU, Intensive Care Unit; HFNO High-flow Nasal Oxygen; PEEP, Positive End Expiratory Pressure; NIV Noninvasive ventilation; RR, Respiratory Rate; SBP, Systolic Blood Pressure; DBP, Diastolic Blood Pressure

^a^Creatinine clearance < 60 mL min^−1^

Level of respiratory support, oxygenation and hemodynamic status were balanced between the two groups at inclusion. More patients allocated to the prone group had HFNO at randomization compared to the control group (86% vs. 74%).

### Primary endpoint

**Fig. 2:**
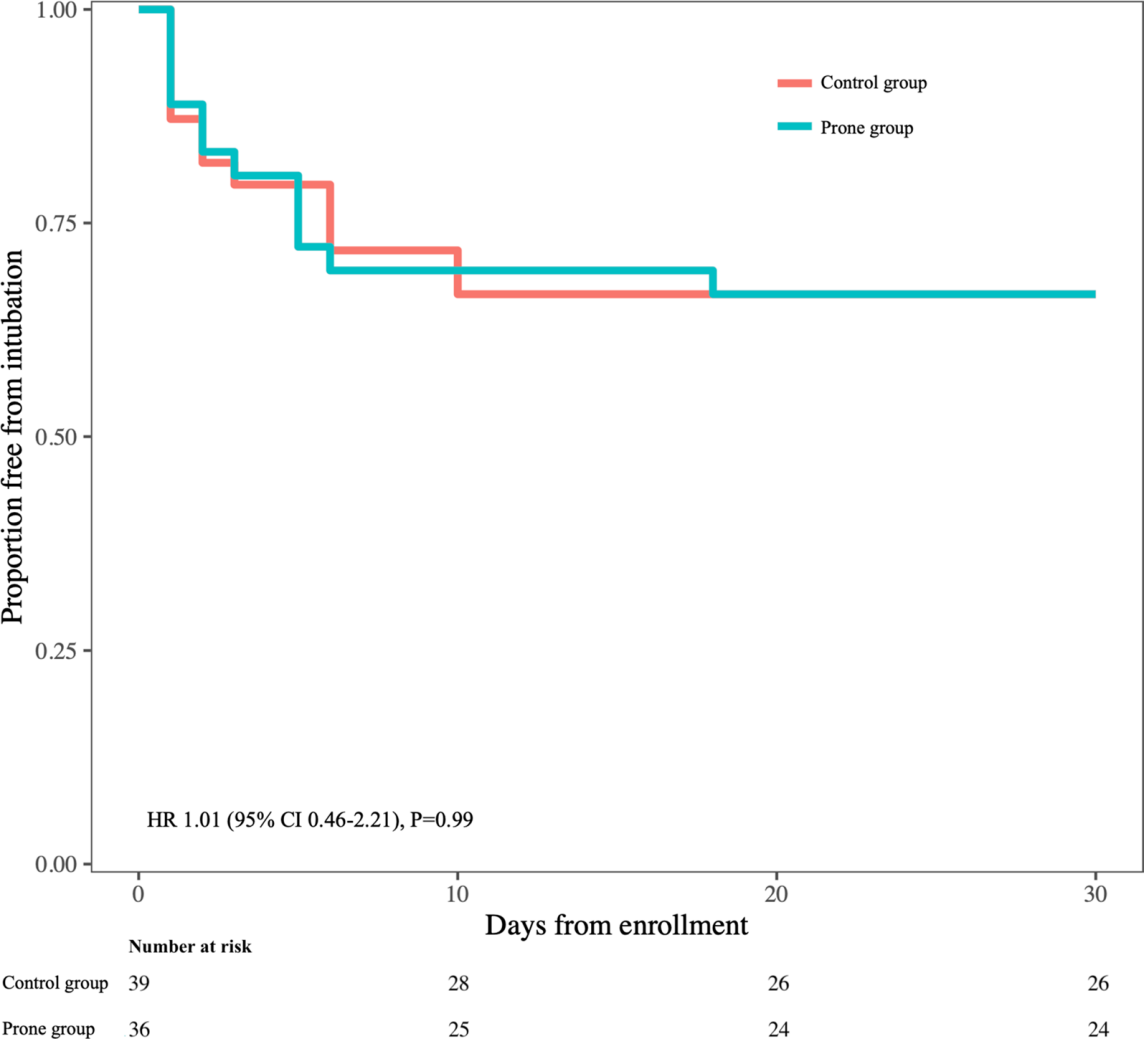
Kaplan–Meier survival analysis. Within 30 days, 13 patients (33%) were intubated in the control group compared with 12 patients (33%) in the prone group, HR 1.01 (95% CI 0.46–2.21), *P* = 0.99

Within 30 days after enrollment, 13 patients (33%) in the control group and 12 patients (33%) in the prone group were intubated (HR 1.01 (95% CI 0.46–2.21), *P* = 0.99) (Fig. 2).

### Secondary endpoints

Duration of early APP (first three days after enrollment) and total APP (all days from enrollment to protocol discontinuation) was longer in the prone group compared with the control group (Table 2). In the prone group, the duration of APP was ≥ 16 h for five (14%) patients during the first day in protocol, and the mean duration of APP was ≥ 16 h for two (6%) patients for all days in protocol.

**Table 2:** Secondary outcomes for the study cohort

Variable	Control group	Prone group	*P* value
Count	39	36	
Daily total prone time, hours	3.4 [1.8–8.4]	9.0 [4.4–10.6]	0.014
Total protocol duration, days	4.9 [2.3–8.1]	4.2 [1.7–5.7]	0.33
Daily prone time day 1–3, hours	2.6 [0.3–8.1]	8.5 [5.2–12.2]	0.001
30-day mortality	3 (8%)	6 (17%)	0.30
VFD^a^, all patients, days	30 [11–30]	30 [12–30]	0.69
VFD^a^, intubated patients, days	2 [1–10]	7 [0–20]	0.38
Days free from HFNO/NIV^b^	24 [22–26]	26 [23–28]	0.15
Enrolment to IMV, days	2 [1–6]	2 [1–5]	0.59
Use of NIV	27 (69%)	21 (58%)	0.33
Enrolment to NIV, days	0.25 [0.1–1.1]	0.23 [0.05–1.2]	0.63
Admitted to ICU	27 (69%)	27 (75%)	0.58
ICU LOS, days	11 [3–22]	5 [4–13]	0.25
ICU-free days^c^	26 [8–30]	25 [14–28]	0.56
Hospital LOS, days	18 [11–30]	16 [11–22]	0.44
Vasoactive drugs	17 (44%)	13 (37%)	0.57
Sedation by continuous infusion^d^	14 (36%)	16 (44%)	0.45
Renal replacement therapy	1 (3%)	1 (3%)	–
ECMO	1 (3%)	0 (0%)	–
WHO clinical progression scale day 7, (0–10)	6 [6, 7]	6 [5–7]	0.35
WHO clinical progression scale, day 30, (0–10)	2 [2–6]	2 [2–4]	0.28
Adverse events			
Pressure sores	9 (23%)	2 (6%)	0.032
Vomiting during proning	0 (0%)	1 (3%)	-
Central or arterial line dislodgement	0 (0%)	0 (0%)	-
Cardiac arrest within 30 days	1 (3%)	2 (6%)	0.51
During proning	0 (0%)	0 (0%)	-

Categorical parameters are presented as *n* (%), continuous variables as median (interquartile range [IQR]), VFD, Ventilator-Free Days; HFNO High-flow Nasal Oxygen; NIV, Noninvasive Ventilation; IMV, Invasive Mechanical Ventilation; ICU, Intensive Care Unit; LOS, Length of Stay; ECMO, Extracorporeal Membrane Oxygenation; WHO, World Health Organization

^a^Ventilator-free days were calculated for intubated (Control *n* = 13, Prone *n* = 12) and all patients, respectively, and defined as days free from invasive mechanical ventilation from enrollment until day 30. Patients who died were registered as 0 VFD

^b^Patients who were not intubated. Calculated from enrollment until day 30. Control *n* = 26, Prone *n* = 24

^c^Days when patients were not in the ICU. Patients who died were registered as 0 ICU-free days

^d^Non-intubated patients during protocol

Three patients (8%) died in the control group compared with six patients (17%) in the prone group (HR 2.29 (95% CI 0.57–9.14), *P* = 0.30). There were no significant differences between groups regarding ventilator-free days for intubated patients, days free of NIV/HFNO for patients not intubated, hospital or ICU length of stay or use of organ support between groups. A majority of patients in both groups received respiratory support with NIV within one day after enrollment.

### Adverse events

Nine patients (23%) in the control group had pressure sores, all located in the lower back or gluteal region, compared with two patients (6%) in the prone group that were both related to pressure from the HFNO (difference − 18% (95% CI − 2 to − 33%); *P* = 0.032). Three cardiac arrests occurred, one in the control group and two in the prone group but none related to APP.

### Exploratory analysis

Patients with duration of APP shorter than 3 h (*n* = 26) versus longer than 9 h (*n* = 26) irrespective of allocation (median prone duration 0.46 [IQR 0–2.2] versus 11.9 [IQR 10.4–13.5] h per day, *P* ≤ 0.001) were compared using Cox’s proportional hazards model, but there was no significant difference in the proportion of patients being intubated in unadjusted analysis (HR 1.14 (95% CI 0.44–2.96), *P* = 0.79) or in analysis adjusted for age and PaO_2_/FiO_2_ at enrollment (HR 0.79 (95% CI 0.29–2.18), *P* = 0.65) (Additional file 1: eFigure 3).

Sub-analysis of patients with PaO_2_/FiO_2_ ratio ≤ 15 kPa did not show any difference in the proportion of patients being intubated between groups in unadjusted analysis (HR 0.94 (95% CI 0.35–2.50), *P* = 0.90) or when adjusting for age (HR 0.51 (95% CI 0.25–1.89), *P* = 0.49). Among patients in this sub-cohort, median prone duration per day was 3.8 h [IQR 2.0–6.5] in the control group (*n* = 13) compared with a median of 8.5 h [IQR 6.5–10.8] in the prone group (*n* = 14), *P* = 0.021 (Additional file 1: eFigure 4).

## Discussion

This is to the best of our knowledge the first randomized clinical trial investigating prolonged prone positioning in non-intubated spontaneously breathing patients with COVID-19. The main finding was that implementation of a protocol for APP increased the duration of prone positioning but did not affect the rate of intubation in patients with moderate to severe hypoxemic respiratory failure compared with standard care. However, only a minority of patients in the prone group complied with the protocol target of 16 h APP duration per day. Furthermore, there were no statistically significant differences in the use of other supportive treatments, 30-day mortality or time to recovery, although these analyses may have been underpowered.

The results of this study were consistent also in exploratory *post-hoc* analyses subgrouping patients according to the duration of APP irrespective of group allocation. Further, no benefit of prolonged APP was found in patients with PaO_2_/FiO_2_ ratio < 15 kPa at inclusion between the prone and control group.

Prone positioning in mechanically ventilated patients with COVID-19 improves oxygenation and is associated with reduced mortality [33]. Although APP similarly improves oxygenation in non-intubated patients with COVID-19 [3, 16–23], reports have failed to show benefits on patient-centered outcomes [25, 26]. A multicenter observational study, investigating a cohort of 199 patients with COVID-19 found no difference in intubation rates in patients with duration of APP for more than 16 h per day compared with shorter duration of APP [25]. They reported similar baseline characteristics, degree of respiratory failure and mortality but higher intubation rates (41% in the control group and 40% in the prone group) compared with our investigation. Further corroborating our results, a single center observational study including 166 patients with COVID-19 with respiratory rate ≥ 24/min who required oxygen supplementation ≥ 3 L min^−1^ found no difference in intubation rates or ICU admission in patients who were treated with APP compared to those who were not [26]. Although the patients in this study were younger and had less severe respiratory failure at inclusion compared to our population, the authors reported higher overall intubation rates (58% in the prone group and 49% in the control group) compared with our trial.

There are several possible explanations for the neutral result of our investigation. Due to observed beneficial physiological effects, patients with COVID-19 were increasingly treated with APP as part of standard care during the study period at the participating study hospitals, resulting in longer APP duration than expected in the control group. The optimal duration of prone positioning is unknown. However, the mean duration of prone positioning was 17 h per day in the prone group compared to 0 h in the supine group in the first study that reported mortality benefit in mechanically ventilated patients [1]. Although the median duration of APP per day was 9.0 h in the prone group compared with 3.4 h in the control group in our study, this difference may not have been large enough to decrease the rate of intubation. Intubated patients are often heavily sedated to tolerate prone positioning and it may be difficult to reach a similar duration of prone positioning in awake patients. Our protocol targeted an APP duration of 16 h in the prone group, but only two (6%) patients were able to reach this target. This is similar to a previous study [23] and indicates that treatment adherence is a major limitation of APP.

Reduction in lung injury associated with mechanical ventilation may in part explain the mortality benefit in mechanically ventilated ARDS [1] and COVID-19 patients [33] undergoing prone positioning [34]. In non-intubated critically ill COVID-19 patients, APP may delay intubation due to temporary improvements in oxygenation [25] which could paradoxically lead to patient self-inflicted lung injury (P-SILI) [35, 36]. Although not statistically significant, there were more deaths in the prone group compared with controls raising concerns of harm associated with awake prone positioning. However, time to intubation was similar between groups, and P-SILI thus may not be a relevant mechanism contributing to mortality in our population. A recently published meta-analysis of non-randomized cohort studies found no difference in mortality or duration of invasive mechanical ventilation in critically ill patients with COVID-19 who were intubated early compared with patients who were intubated late [37]. Although further research is warranted, this could suggest that harm associated with early intubation outweighs harm associated with P-SILI in delayed intubation or that P-SILI may not be an important mechanism in COVID-19 [38].

Among intubated patients in our study, there were more ventilator-free days in the prone group compared with the control group and among non-intubated patients there were more days free from NIV/HFNO in the prone group compared with the control group. These differences were not statistically significant, possibly due to low statistical power. Reducing duration of invasive and noninvasive respiratory support is important, in particular in settings with resource shortage. Ventilator-free days or days free from any respiratory support including NIV and HFNO [39] may thus be an appropriate primary outcome of future studies.

Contrary to intubated patients, where prone position increases the risk of pressure sores [40], patients in the prone group had fewer pressure sores than patients in the control group in our study. Frequent changes in body position may have reduced the risk of lower back and gluteal pressure sores in the prone group. Pressure sores have been associated with higher mortality in ICU patients and constitute a considerable burden to healthcare systems [41]. The large number of patients during the pandemic combined with the high proportion of pressure sores in the control group indicates that this may be a substantial problem and the present investigation highlights the need for protocolized mobilization in critically ill patients.

Strengths of the present study included the randomized multicenter design and the well-defined protocol for APP increasing generalizability and reproducibility. This trial was conducted during the second pandemic wave, and physicians, nurses and physiotherapists at the participating ICUs and wards gained extensive experience of prone positioning in non-intubated patients during the first wave, ensuring high-quality APP for included patients. No patients were lost to follow up and there was minimal missing data. As the first randomized clinical trial of prolonged APP in COVID-19, this trial provides important new information to bedside clinicians and for future studies.

There are also limitations to this trial. First, the trial was halted early resulting in limited statistical power to detect differences between groups. In particular, analyses of subgroups that may benefit from APP and analyses of secondary outcomes with few events may have been hampered and the results should therefore be interpreted with caution. Second, due to the nature of the intervention, blinding was not possible, increasing the risk of bias. Third, we included patients with moderate to severe respiratory failure and there was a liberal use of NIV in both groups early after enrollment. Our results may therefore not be generalizable to patients with less severe degrees of respiratory failure and settings where prolonged respiratory support with HFNO is standard care. Fourth as all study sites became overwhelmed by severely ill patients with COVID-19, and research staff was relocated for clinical service, we were not able to identify all patients eligible for inclusion. Fifth, APP was increasingly considered standard of care in COVID-19-related hypoxemic respiratory failure attenuating the difference in duration of APP between groups. Finally, we did not pre-define criteria for intubation or de-escalation from NIV/HFNO to low flow oxygen therapy. This was a pragmatic choice to minimize extra workload on clinicians and decrease risk of protocol compliance issues.

## Conclusions

The implemented protocol for APP and standard care among patients with hypoxemic respiratory failure due to COVID-19 was safe and increased the duration of prone position, but did not reduce the rate of endotracheal intubation compared with standard care alone.

## Supplementary Information

**Additional file 1.** Supplementary information. **eTable 1**. Inclusion criteria based on SpO_2_ at various FiO_2_-values approximating a PaO_2_/FiO_2_ ratio of 20 kPa (150 mmHg). **eTable 2**. Interim analysis criteria for early trial termination. **eFigure 1**. Trial protocol flow-chart. **eFigure 2a-c**. Trial definitions of prone and semi-prone position. **eFigure 3**. Results of the exploratory analysis of patients with shorter (n = 26) than 3 h or longer (n = 26) than 9 h duration of awake prone positioning irrespective of group allocation presented as Kaplan-Meier graphs. **eFigure 4**. Results of the exploratory analysis of patients in the control group (n = 13) and prone group (n = 14) with PaO_2_/FiO_2_ ratio ≤ 15 kPa presented as Kaplan-Meier graphs.

## Data Availability

The datasets used and/or analyzed during the current study are available from the corresponding author on reasonable request.

## Abbreviations

APP: Awake prone positioning
ARDS: Acute respiratory distress syndrome
CRRT: Continuous renal replacement therapy
COVID-19: Coronavirus disease 2019
ECMO: Extracorporeal membrane oxygenation
HFNO: High-flow nasal oxygen
ICU: Intensive care unit
IQR: Interquartile range
NIV: Noninvasive ventilation
PEEP: Positive end expiratory pressure
P-SILI: Patient self-inflicted lung injury
