# Supplementary material for: Awake prone positioning in patients with hypoxemic respiratory failure due to COVID-19: the PROFLO multicenter randomized clinical trial
Source: Crit Care. 2021 Jun 14;25:209. doi: 10.1186/s13054-021-03602-9 (PMC8200797; doi:10.1186/s13054-021-03602-9)

Additional File 1

eTable 1 2

eTable 2 3

eFigure 1 4

eFigure 2a-c 5

eFigure 3 6

eFigure 4 7

# eTable 1. Inclusion criteria based on SpO_2_ at various FiO_2_-values approximating a PaO_2_/FiO_2_ ratio of 20 kPa (150 mmHg).

| **FiO_2_** | **SpO_2_** |
| --- | --- |
| 0.40 | ≤ 90% |
| 0.45 | ≤ 92% |
| 0.50 | ≤ 94% |
| 0.55 | ≤ 96% |
| 0.60 | ≤ 98% |

**eTable 2.** Interim analysis criteria for early trial termination.

| **Criteria** | **Definition** |
| --- | --- |
| Futility | Lack of effect or lack of patients fulfilling the inclusion criteria |
| Safety | Unexpected increase in the rate of intubation in the intervention group or increased occurrence of severe or unexpected adverse events. |
| Efficacy | If the reduction in the rate of intubation is more than 40% |

# eFigure 1. Trial protocol flow-chart.

# *COVID-19: Coronavirus disease 2019. HFNO: High-flow nasal oxygen. APP: Awake prone positioning.*

**eFigure 2a-c.** Trial definitions of prone and semi-prone position.

**eFigure 2a.** Prone position version 1.


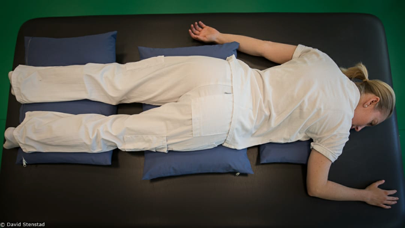


**eFigure 2b.** Prone position version 2.


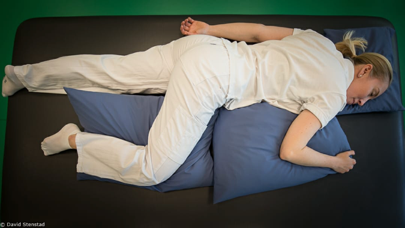


**eFigure 2c.** Semi-prone position.
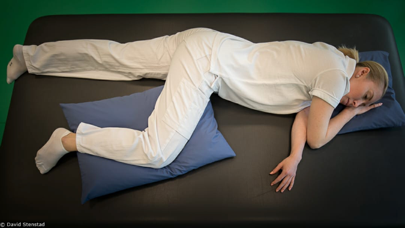


**eFigure 3.** Results of the exploratory analysis of patients with shorter (n=26) than 3 hours or longer (n=26) than 9 h duration of awake prone positioning irrespective of group allocation presented as Kaplan-Meier graphs.


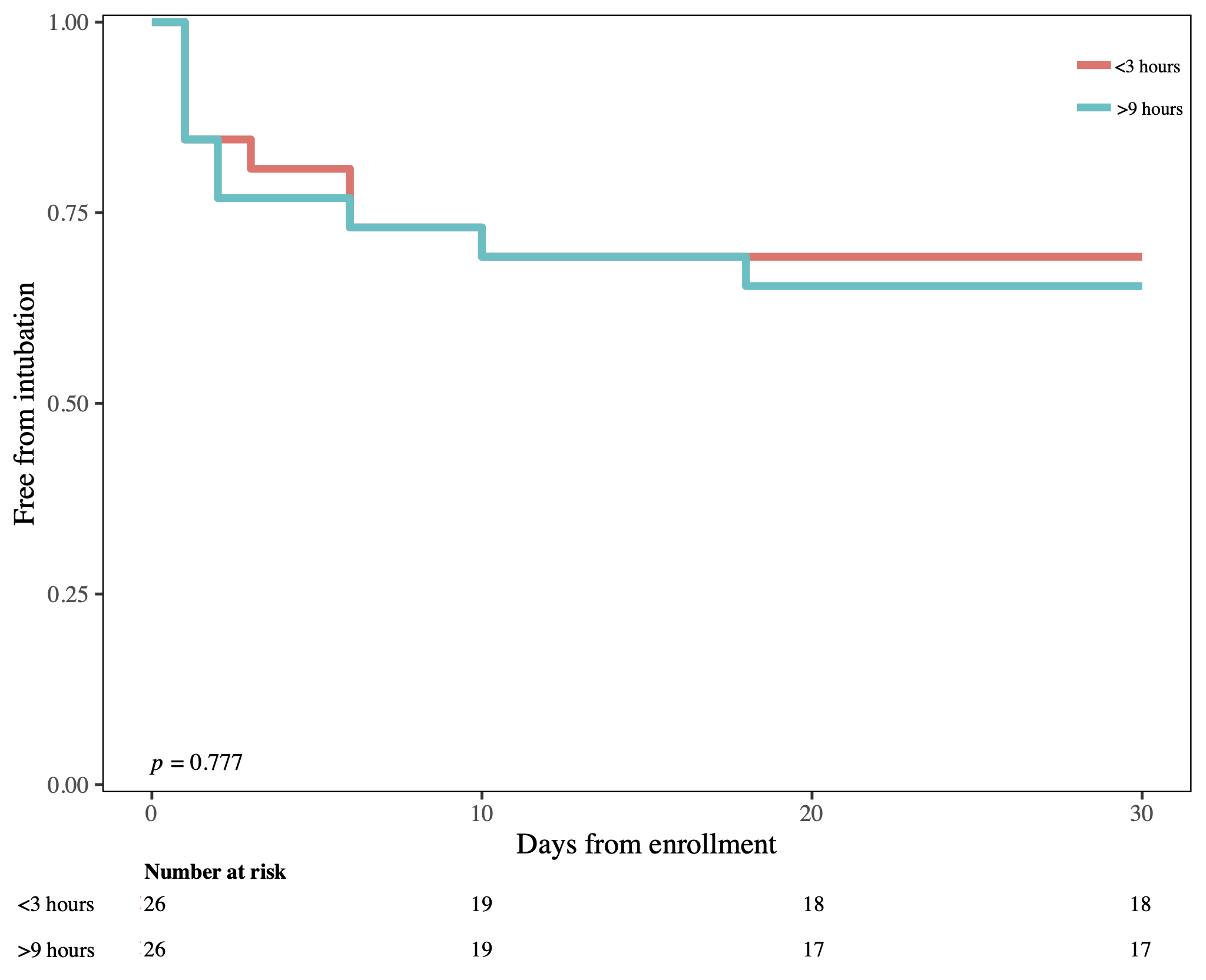


**eFigure 4.** Results of the exploratory analysis of patients in the control group (n=13) and prone group (n=14) with PaO2/FiO2 ratio ≤ 15 kPa presented as Kaplan-Meier graphs.


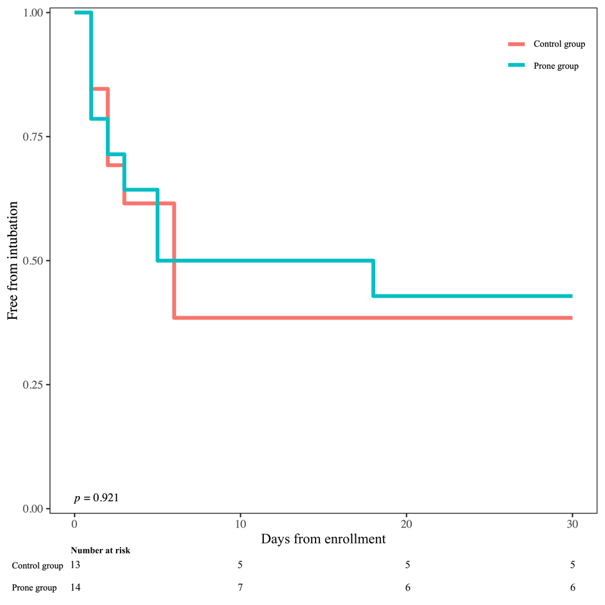

Supplement: Supplementary file 1 — Additional file 1. Supplementary information. eTable 1. Inclusion criteria based on SpO2 at various FiO2-values approximating a PaO2/FiO2 ratio of 20 kPa (150 mmHg). eTable 2. Interim analysis criteria for early trial termination. eFigure 1. Trial protocol flow-chart. eFigure 2a-c. Trial definitions of prone and semi-prone position. eFigure 3. Results of the exploratory analysis of patients with shorter (n = 26) than 3 h or longer (n = 26) than 9 h duration of awake prone positioning irrespective of group allocation presented as Kaplan-Meier graphs. eFigure 4. Results of the exploratory analysis of patients in the control group (n = 13) and prone group (n = 14) with PaO2/FiO2 ratio ≤ 15 kPa presented as Kaplan-Meier graphs. [file 13054_2021_3602_MOESM1_ESM.docx]
